# Population Effectiveness of Dolutegravir Implementation in Uganda: A Prospective Observational Cohort Study (DISCO), 48-Week Results

**DOI:** 10.1093/infdis/jiae260

**Published:** 2024-05-16

**Authors:** Suzanne M McCluskey, Winnie R Muyindike, Victoria Nanfuka, Daniel Omoding, Nimusiima Komukama, Ian T Barigye, Lydia Kansiime, Justus Tumusiime, Taing N Aung, Ashley Stuckwisch, Bethany Hedt-Gauthier, Vincent C Marconi, Mahomed-Yunus S Moosa, Deenan Pillay, Jennifer Giandhari, Richard Lessells, Ravindra K Gupta, Mark J Siedner

**Affiliations:** Medical Practice Evaluation Center, Massachusetts General Hospital, Boston, Massachusetts, USA; Division of Infectious Diseases, Massachusetts General Hospital, Boston, Massachusetts, USA; Harvard Medical School, Boston, Massachusetts, USA; Mbarara University of Science and Technology, Mbarara, Uganda; Mbarara University of Science and Technology, Mbarara, Uganda; Mbarara University of Science and Technology, Mbarara, Uganda; Mbarara University of Science and Technology, Mbarara, Uganda; Mbarara University of Science and Technology, Mbarara, Uganda; Mbarara University of Science and Technology, Mbarara, Uganda; Mbarara University of Science and Technology, Mbarara, Uganda; Medical Practice Evaluation Center, Massachusetts General Hospital, Boston, Massachusetts, USA; Medical Practice Evaluation Center, Massachusetts General Hospital, Boston, Massachusetts, USA; Harvard Medical School, Boston, Massachusetts, USA; Biostatistics, Harvard T. H. Chan School of Public Health, Boston, Massachusetts, USA; Emory University School of Medicine and Rollins School of Public Health, Emory University, Atlanta, Georgia, USA; Department of Infectious Diseases, University of KwaZulu-Natal, Durban, South Africa; Division of Infection and Immunity, University College London, London, United Kingdom; KwaZulu-Natal Research Innovation and Sequencing Platform, Nelson R. Mandela School of Medicine, University of KwaZulu-Natal, Durban, South Africa; Department of Infectious Diseases, University of KwaZulu-Natal, Durban, South Africa; Cambridge Institute of Therapeutic Immunology & Infectious Diseases, University of Cambridge, Cambridge, United Kingdom; Africa Health Research Institute, Durban, South Africa; Medical Practice Evaluation Center, Massachusetts General Hospital, Boston, Massachusetts, USA; Division of Infectious Diseases, Massachusetts General Hospital, Boston, Massachusetts, USA; Harvard Medical School, Boston, Massachusetts, USA; Africa Health Research Institute, Durban, South Africa

**Keywords:** HIV drug resistance, antiretroviral therapy, dolutegravir, TLD, sub-Saharan Africa

## Abstract

**Background:**

Tenofovir/lamivudine/dolutegravir (TLD) is the preferred first-line antiretroviral therapy (ART) regimen for people with HIV (PWH), including those who were previously virologically suppressed on nonnucleoside reverse transcriptase inhibitors (NNRTIs). We sought to estimate the real-world effectiveness of the TLD transition in Ugandan public-sector clinics.

**Methods:**

We conducted a prospective cohort study of PWH aged ≥18 years who were transitioned from NNRTI-based ART to TLD. Study visits were conducted on the day of TLD transition and 24 and 48 weeks later. The primary end point was viral suppression (<200 copies/mL) at 48 weeks. We collected blood for retrospective viral load (VL) assessment and conducted genotypic resistance tests for specimens with VL >500 copies/mL.

**Results:**

We enrolled 500 participants (median age 47 years; 41% women). At 48 weeks after TLD transition, 94% of participants were in care with a VL <200 copies/mL (n = 469/500); 2% (n = 11/500) were lost from care or died; and only 2% (n = 9/500) had a VL >500 copies/mL. No incident resistance to DTG was identified. Few participants (2%, n = 9/500) discontinued TLD due to adverse events.

**Conclusions:**

High rates of viral suppression, high tolerability, and lack of emergent drug resistance support use of TLD as the preferred first-line regimen in the region.

**Clinical Trials Registration:**

NCT04066036.

## Teaser

Among people with HIV in Uganda who transitioned to TLD, we observed high rates of viral suppression, high tolerability, and no emergent drug resistance, all of which support use of TLD as the preferred first-line regimen in the region.

Over the past 5 years, a global paradigm shift in human immunodeficiency virus (HIV) treatment guidelines has occurred such that over 18 million people are now on antiretroviral therapy (ART) containing the integrase strand transfer inhibitor dolutegravir (DTG) [1]. This updated recommendation was made by the World Health Organization (WHO) in 2018 in response to rising rates of pretreatment drug resistance to nonnucleoside reverse transcriptase inhibitors (NNRTIs) [2, 3] and introduction of a generic single-tablet combination of tenofovir, lamivudine, and DTG (known as TLD), which was less costly than previous first-line ART regimens [4]. TLD was prioritized for both individuals newly initiating ART and for those who were already on NNRTI-containing first-line regimens, given its high genetic barrier to resistance and expected favorable tolerability profile in comparison to NNRTIs [4, 5]. Uganda began the programmatic implementation of TLD in 2018, requiring documentation of viral suppression to <1000 copies/mL within 6 months prior to transition [6]. Initial guidelines for TLD restricted its use among women of child-bearing potential due to historical concerns about neural tube defects, but this restriction was lifted in the 2020 update to the Uganda HIV management guidelines when additional data on its safety emerged [6, 7].

Although DTG has a high genetic barrier to resistance [8, 9], virologic failure and treatment-emergent resistance have been demonstrated in DTG monotherapy studies [10, 11]. Furthermore, dual class resistance to both NNRTIs and nucleoside reverse transcriptase inhibitors (NRTIs) was common in the region among those with virologic failure on NNRTI-containing first-line regimens [12, 13]. Although concerns about the latter have been partially eased by results of the NADIA trial, the few individuals who did develop integrase inhibitor resistance in that study had preexisting resistance to NRTIs [14, 15]. Furthermore, pretreatment NNRTI resistance has also been associated with compromised virologic responses to first-line DTG-based ART in South Africa [16]. Thus, theoretical concerns remain regarding how TLD will perform in the setting of prior treatment experience, particularly when used at scale in millions of people in the public sector of sub-Saharan Africa, where prior regimens with high barriers to resistance have also failed to sustain full activity [17]. The potential for this scenario increases in settings for which viral suppression thresholds prior to TLD transition are less restrictive and in settings that do not require a viral load prior to TLD transition, particularly given prior associations between viral load monitoring and prevalence of drug resistance following virologic failure on NNRTI-containing first-line regimens [18].

Our objective was to conduct a prospective longitudinal cohort study to examine the durability of viral suppression, rates of treatment-emergent resistance, and tolerability of TLD in the public sector in Uganda.

## METHODS

### Study Design and Participants

We conducted a prospective cohort study in southwestern Uganda from May 2019 to 2021 (NCT04066036). We enrolled adults with HIV who were at least 18 years old and were programmatically transitioned from NNRTI-containing first-line ART to TLD by the clinic staff. Individuals were eligible for the study who had been on ART for at least 6 months, were enrolled in care at Mbarara Regional Referral Hospital Immune Suppression Syndrome Clinic, resided within 100 kilometers of the clinic, and intended to remain in the catchment area for the duration of the follow-up period.

### Study Procedures

We enrolled participants on the date that TLD was first prescribed to them by the clinic. Study visits occurred on the date of enrollment and at 24 and 48 weeks after TLD transition. We defined 24-week follow-up visits as any visit occurring 20–44 weeks after TLD transition and prior to eligibility for the 48-week visit. We defined the 48-week follow-up visit as any study visit occurring from 44 weeks after TLD transition up until the close of data collection for 48-week visits for the entire study. Visits were coordinated with participants’ planned clinic visits when feasible. At each study visit, we administered questionnaires to obtain information on ART adherence, concurrent medication use, symptoms, sleep, and pregnancy history. We conducted a chart review at each study visit to obtain data on the participants’ HIV and ART history and documented laboratory studies. We collected blood at each visit, which was processed and stored at −80°C until the time of testing. For visits that occurred during the coronavirus disease 2019 (COVID-19) pandemic (years 2020–2021), questionnaires were administered by phone, and participant specimens were obtained during routine clinic appointments. We employed phone tracking for participants who missed study follow-up visits. If a participant missed the 48-week study visit, home tracking visits were conducted if the participant had consented to this at the time of enrollment.

### Laboratory Procedures

We measured HIV-1 RNA viral loads (VL) on plasma specimens from each study visit at the Mbarara University of Science and Technology Clinical and Research Laboratory using the GeneXpert platform (Cepheid). We performed genotypic resistance testing (GRT) of reverse transcriptase and integrase regions of the *pol* gene on all plasma specimens with a VL >500 copies/mL. GRT was conducted at the KwaZulu-Natal Research Innovation and Sequencing Platform in Durban, South Africa using in-house Sanger sequencing. HIV drug resistance mutations were identified using the Stanford algorithm [19, 20].

### Statistical Analysis

We described the study population characteristics using medians with interquartile range (IQR) for continuous variables and proportions for categorical variables. We summarized study outcomes at 48 weeks after TLD transition for the entire study population, categorized as in care with viral suppression, in care and unsuppressed, in care with missing VL data, lost from care, or deceased. We defined viral suppression as study VL <200 copies/mL for the primary outcome of interest and study VL <50 copies/mL as a secondary outcome. We defined retention in care as completion of the 48-week study visit, missed 48-week study visit but with confirmed return to clinical care, or missed 48-week study visit but with confirmed transfer of care to a different clinic. We also described rates of viral suppression at enrollment and 24 weeks.

We fit multivariable logistic regression models to assess predictors of viral suppression with retention in care at 48 weeks, using VL thresholds of both 50 and 200 copies/mL. We also evaluated additional characteristics as potential predictors in univariate models, including age, marital status, education level, reported symptoms while on TLD, ART duration, and time between last clinic VL and TLD transition, none of which were significant predictors of the outcome of interest and were not included in the final models. We used a complete case analysis to account for missing data. All analyses were conducted using Stata 14.0.

### Ethics

All participants consented to participation in the study. The study was approved by institutional review boards at Mass General Brigham, Mbarara University of Science and Technology, and the Uganda National Council of Science and Technology.

## RESULTS

### Participant Characteristics

**Figure 1.**
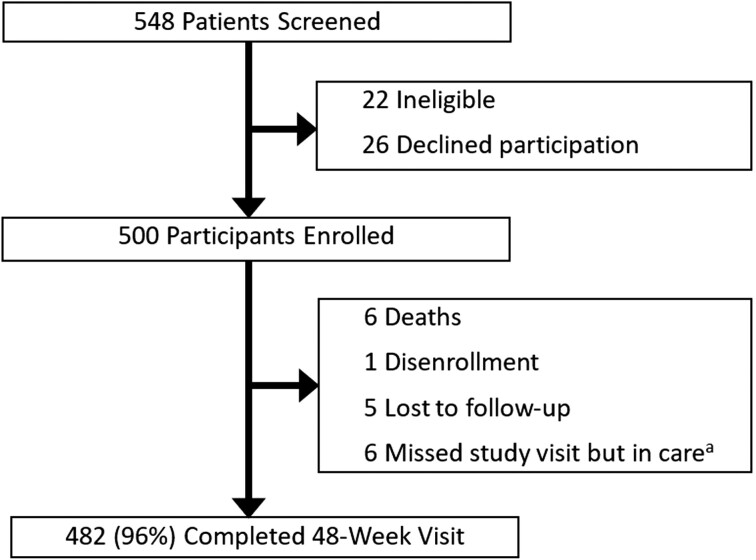
Study flow chart. ^a^This category includes participants who confirmed transfer of care to another clinic or who remained in care at the clinic but did not have 48-week study viral load data available.

Of 548 individuals screened, we enrolled 500 participants who were eligible and consented to participation in the study (Figure 1). Participant characteristics are summarized in Table 1. Forty-one percent (n = 205/500) of the study participants were female, and the median age was 47 years (IQR, 40–53 years). Ninety-nine percent (n = 494/500) of the cohort had been on ART for greater than 1 year at the time of transition to TLD with a median duration of ART prior to transition of 8.8 years (IQR, 5.7–12.2 years). The most common ART regimen prior to transition was lamivudine/tenofovir/efavirenz (44%, n = 222/500), followed by lamivudine/zidovudine/nevirapine (39%, n = 193/500). The median time from the last VL measurement by the clinic prior to TLD transition and the date of TLD transition was 9 weeks (IQR, 8–13 weeks).

**Table 1.** Study Population Characteristics (N = 500)

Characteristics	Value
Age, y, median (IQR)	47 (40–53)
Female, n (%)	205 (41)
Marital status, n (%)	
Not married	18 (3)
Married	318 (64)
Domestic partnership	10 (2)
Separated	39 (8)
Divorced	8 (2)
Widowed	107 (21)
Education level, n (%)	
No schooling	56 (11)
Primary	254 (51)
Secondary	109 (22)
Tertiary	81 (16)
Years on ART, median (IQR)	9 (6–12)
ART regimen prior to TLD, n (%)	
3TC/TDF/EFV	222 (44)
3TC/AZT/NVP	193 (39)
Other	85 (17)

Abbreviations: 3TC, lamivudine; ART, antiretroviral therapy; AZT, zidovudine; EFV, efavirenz; IQR, interquartile range; NVP, nevirapine; TDF, tenofovir; TLD, tenofovir/lamivudine/dolutegravir.

### Participant Outcomes

Ninety percent of those enrolled (n = 448/500) completed 24-week visits, which occurred a median of 24 weeks (IQR, 24–26 weeks) after TLD transition, and 96% (n = 482/500) completed 48-week visits, which occurred a median of 54 weeks (IQR, 49–67 weeks) after TLD transition (Figure 1). Eight percent of 24-week visits and 100% of 48-week visits occurred after transportation restrictions were implemented in response to the COVID-19 pandemic in Uganda in March 2020 [21]. Of those participants who did not complete a 48-week visit, < 1% (n = 1/500) disenrolled, < 1% (n = 1/500) transferred care to another clinic, 1% (n = 5/500) remained in care at the clinic but did not have VL data available, 1% (n = 5/500) were lost from care, and 1% (n = 6/500) of participants died (Figure 1). Causes of death were unspecified respiratory illness (n = 1), leukemia (n = 1), alcohol intoxication (n = 1), febrile illness/presumed malaria (n = 1), and unknown causes (n = 2).

### Virologic Outcomes

**Figure 2.**
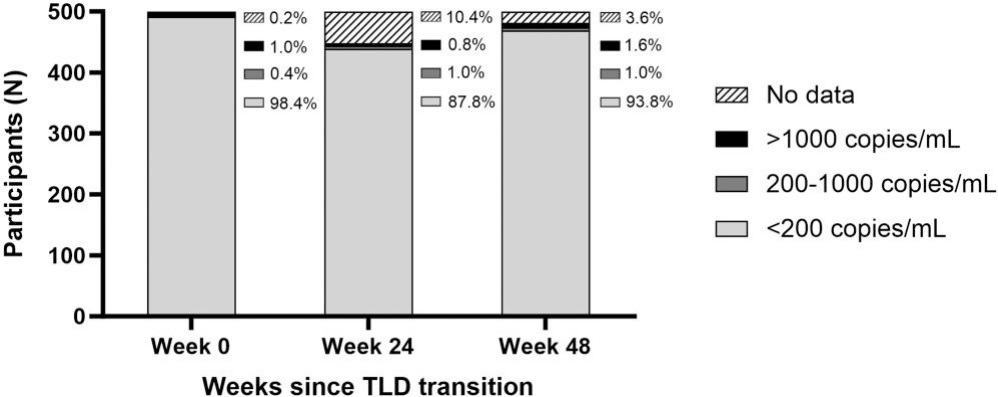
Virologic outcomes on the day of TLD transition, and 24 and 48 weeks after transition to TLD, with viral suppression defined as <200 copies/mL. Abbreviation: TLD, tenofovir/lamivudine/dolutegravir.

At the time of TLD transition/study enrollment, 98% (n = 492/500) were virally suppressed with VL <200 copies/mL, < 1% (n = 2/500) had detectable viremia between 200 and 1000 copies/mL, and 1% (n = 5/500) had a VL >1000 copies/mL (Figure 2). At week 24, 88% (n = 439/500) were virally suppressed to <200 copies/mL, 1% (n = 5/500) had detectable viremia between 200 and 1000 copies/mL, 1% (n = 4/500) had a VL >1000 copies/mL, and 10% (n = 52/500) had missing VL data (Figure 2). At the 48-week study outcome visit, 94% (n = 469/500) were in care and virally suppressed to <200 copies/mL, 1% (n = 5/500) had detectable viremia between 200 and 1000 copies/mL, 2% (n = 8/500) had a VL >1000 copies/mL, 2% (n = 11/500) did not have virologic data due death or loss to follow-up, and 1% (n = 7/500) did not have data due to disenrollment or retention in care with a missed study visit (Figure 1 and Figure 2). When evaluating the secondary end point with viral suppression defined using a VL threshold of <50 copies/mL, 92% (n = 459/500) were in care and virally suppressed. In a multivariable regression model, those who had a detectable VL ≥50 copies/mL at the time of TLD transition (adjusted odds ration [aOR], 0.12; 95% confidence interval [CI], .04–.33), men (aOR, 0.38; 95% CI, .16–.91), and those with self-reported ART adherence <90% at any point during follow-up (aOR, 0.28; 95% CI, .12–.69) were all significantly less likely to achieve viral suppression to <50 copies/mL with retention in care at 48 weeks (Supplementary Table 1). Results were similar when defining viral suppression using a VL threshold of <200 copies (Table 2).

**Table 2.** Multivariable Logistic Regression Model to Assess Predictors of Viral Suppression Defined as <200 Copies/mL After 1 Year of Follow-up in the DISCO Cohort

Predictors	Proportion	aOR	95% CI	*P* Value
HIV-1 RNA viral load at time of switch to TLD				
<200 copies/mL at the time of switch	462/492	Reference		
≥200 copies/mL at the time of switch	6/7	0.18	.02–1.68	.13
Sex				
Female	198/205	Reference		
Male	271/295	0.39	.14–1.07	.067
Self-reported adherence during follow-up				
Adherence ≥90%	426/442	Reference		
Any adherence <90%	43/51	0.21	.08–.53	.001

Abbreviations: aOR, adjusted odds ratio; CI, confidence interval; HIV-1, human immunodeficiency virus-1; TLD, tenofovir/lamivudine/dolutegravir.

### HIV Drug Resistance

Nineteen plasma specimens had a VL >500 copies/mL from enrollment (n = 5), week 24 (n = 5), and week 48 (n = 9) (Table 3). Sanger sequencing of integrase was successful for all 19 specimens but failed for reverse transcriptase in 2 specimens (1 from enrollment and 1 from week 24). GRT results are summarized in Table 3. No integrase mutations were identified in specimens from any time point. Of the 5 enrollment specimens tested, 3 had NRTI mutations. Two of these participants had K65R and M18V mutations, resulting in high-level resistance to tenofovir and lamivudine, and both achieved viral suppression to <50 copies/mL after transition to TLD. Of the 5 participants with GRT results from week 24, none had viremia at the time of enrollment. One participant was found to have M184V and resuppressed by week 48. At 48 weeks, the majority of sequenced specimens were found to be wild-type HIV-1. One participant was found to have NRTI mutations K70E, M184V, and K219R resulting in low-level resistance to tenofovir and high-level resistance to lamivudine, as well as resistance to NNRTIs. However, this participant had previously been suppressed on an NNRTI-containing regimen and did not develop integrase resistance. All participants with follow-up specimens that were sequenced were on TLD. None of the participants whose specimens were sequenced during follow-up had detectable viremia at earlier time points in the study. All participants with VL >500 copies/mL at 24 weeks resuppressed by week 48.

**Table 3.** HIV Drug Resistance Outcomes

Participant	Week 0 VL, copies/mL	Week 0 HIVDR Mutations			Week 24 VL, copies/mL	Week 24 HIVDR Mutations			Week 48 VL, copies/mL	Week 48 HIVDR Mutations		
		NRTI	NNRTI	INSTI		NRTI	NNRTI	INSTI		NRTI	NNRTI	INSTI
A	2860	E44D, K65R, M184 V, K219Q	K103N, Y181C	None	<40	NA	NA	NA	<40	NA	NA	NA
B	5860	K65R, D67G, M184MV, K219E	V106A, G190V	None	<40	NA	NA	NA	<40	NA	NA	NA
C	10 700	None	E138A	None	<40	NA	NA	NA	320	NA	NA	NA
D	2900	Failed RT sequencing	Failed RT sequencing	None	MV	MV	MV	MV	<40	NA	NA	NA
E	6840	M184V	K103N,V108I	None	MV	MV	MV	MV	<40	NA	NA	NA
F	<40	NA	NA	NA	19 100	None	E138EA	None	<40	NA	NA	NA
G	<40	NA	NA	NA	1440	None	None	None	<40	NA	NA	NA
H	<40	NA	NA	NA	12 200	None	None	None	<40	NA	NA	NA
I	56	NA	NA	NA	640	Failed RT sequencing	Failed RT sequencing	None	<40	NA	NA	NA
J	<40	NA	NA	NA	6700	M184MV	K103KN, V179VAIT, G190GRS	None	<40	NA	NA	NA
K	<40	NA	NA	NA	<40	NA	NA	NA	78 200	None	None	None
L	<40	NA	NA	NA	MV	MV	MV	MV	506 000	None	None	None
M	<40	NA	NA	NA	MV	MV	MV	MV	105 000	None	K103N	None
N	<40	NA	NA	NA	<40	NA	NA	NA	14 800	None	None	None
O	<40	NA	NA	NA	<40	NA	NA	NA	516	None	K103N	None
P	<40	NA	NA	NA	MV	MV	MV	MV	258 000	None	None	None
Q	<40	NA	NA	NA	<40	NA	NA	NA	1 580 000	None	None	None
R	<40	NA	NA	NA	<40	NA	NA	NA	298 000	None	None	None
S	<40	NA	NA	NA	MV	MV	MV	MV	11 000	K70E, M184 V, K219R	A98G, K103N, P225H, F227L	None

Abbreviations: HIV, human immunodeficiency virus; HIVDR, HIV drug resistance; INSTI, integrase strand transfer inhibitor; NA, not applicable; NNRTI, nonnucleoside reverse transcriptase inhibitor; NRTI, nucleoside reverse transcriptase inhibitor; RT, reverse transcriptase; VL, HIV-1 RNA load; MV, missed visit.

### Safety and Tolerability

TLD was discontinued and not restarted in 2% of participants (n = 9/500). Reasons for TLD discontinuation included hyperglycemia (grade 2, n = 1; grade 3, n = 2; grade 4, n = 1), a constellation of symptoms including headache, polyuria, dizziness, and decreased appetite (n = 1), a constellation of symptoms including headache dizziness, paresthesias, poor sleep, and joint pains (n = 1), psychosis (n = 1), concern for a drug-drug interaction (n = 1), and unknown reasons (n = 1). No participants were pregnant at the time of study enrollment or became pregnant during the 48-week follow-up period.

## DISCUSSION

In this prospective cohort of ART-experienced adults who were transitioned from NNRTI-containing regimens to TLD, viral suppression rates exceeded 90% 48 weeks after transition with high rates of retention in care in this public sector clinic. Taken together with no emergent resistance to DTG and high tolerability of TLD, our study results affirm the promise of TLD to revolutionize care and viral suppression rates in the region.

When evaluating outcomes after transition to TLD, 94% of participants were virally suppressed to <200 copies/mL and in care at 48 weeks, while 3% had viremia >200 copies/mL, and 2% died or were lost from care. Those with <90% self-reported adherence during the follow-up period were significantly less likely to achieve viral suppression. In addition, when applying a stricter VL threshold of <50 copies/mL to define viral suppression, men and individuals with detectable viremia at the time of TLD transition were also less likely to achieve viral suppression with retention in care at 48 weeks. Reassuringly, no individuals with viremia at any time point in the study had treatment-emergent resistance to integrase strand transfer inhibitors, including among 2 individuals with preexisting resistance to lamivudine and tenofovir. These results underscore the role of incomplete adherence to ART as the primary driver of virologic failure for individuals on TLD. This contrasts to high rates of HIV drug resistance that have been observed in the context of virologic failure on NNRTI-containing regimens, which have a much lower genetic barrier to resistance [12, 22].

While several clinical trials have evaluated the efficacy of DTG-containing regimens for ART-naive individuals [23, 24], as well as for individuals with virologic failure on NNRTI-containing regimens in sub-Saharan Africa [14, 25–29], this study is among the first to report results from a well-characterized longitudinal cohort of adults in East Africa who were programmatically transitioned to first-line TLD in a public-sector clinic [30, 31]. In addition, few observational studies monitoring the TLD transition in the region have systematically employed GRT to date.

Viral suppression rates on TLD in this study are in line with what has been reported from other observational studies conducted in East Africa. The AFRICOS study, conducted in Uganda, Kenya, Tanzania, and Nigeria, reported a 94% viral suppression rate amongst individuals who were transitioned to TLD [30]. Similarly, data from the International Epidemiology Databases to Evaluate AIDS (IeDEA) Central Africa and East Africa regions revealed a crude incidence rate of 1.5 cases of viremia >1000 copies/mL per 100 person years while on TLD [31].

When comparing our drug resistance outcomes to other studies, data are similarly reassuring for a very low prevalence or even absence of treatment-emergent resistance to DTG among individuals who transitioned to TLD from NNRTI-containing regimens. A national cross-sectional study in Tanzania using WHO acquired drug resistance surveillance methodology identified major INSTI mutations in only 1 out of 41 adults with VLs >1000 copies/mL while on a DTG-containing regimen during the 3-month surveillance period [32]. In addition, a study in Malawi identified DTG resistance in 0.1% of participants with at least 2 VL tests after transition to TLD, and studies in Lesotho and Cameroon found no treatment-emergent resistance to DTG [33–35]. While only 2 individuals in our cohort were found to have high-level resistance to lamivudine and tenofovir at the time of TLD transition, both achieved viral suppression on TLD, thus supporting findings from recent clinical trials highlighting the effectiveness of DTG, even in the setting of resistance to the NRTI backbone [14, 28, 29]. The study from Malawi also reported no increased risk of viremia among those with resistance to the NRTI backbone as compared to a susceptible NRTI backbone; however, it was notable that the 2 participants with treatment-emergent DTG failure in that study were found to have baseline resistance to lamivudine and tenofovir. However, newer data are emerging from the region suggesting higher rates of resistance to DTG over time and particularly in those with a history of virologic failure on other regimens [36, 37]. Future studies with a longer duration of follow-up, as well as systematic drug resistance surveillance, will be needed to either ease concerns more definitively or to raise alarms regarding the emergence of DTG resistance globally.

In addition, while we observed that the great majority of individuals were virally suppressed on the day of TLD transition from a NNRTI-containing regimen (as recommended in Ugandan guidelines [6]), 5% of participants did have occult viremia on the day of TLD transition. Despite reassuring findings regarding the robustness of DTG in the setting of NRTI resistance, our results show that viremia at the time of TLD transition is a risk factor for viremia while on TLD during follow-up, which has also been raised by other studies [31, 33]. This reinforces that preexisting adherence barriers, rather than resistance, may be the primary contributor to viremia while on TLD. Drug-level studies are planned to further evaluate this hypothesis within the DISCO cohort.

Results of this study should be interpreted in light of limitations. These data were collected from a single public sector clinic in Uganda, which is a regional referral center, and may not be applicable to more remote centers or those in other regions. In addition, we report follow-up over a 48-week time period, and a longer duration of follow-up may be needed to identify virologic failure and drug resistance for those on DTG-based regimens. Of note, this cohort was composed of 41% women, which differs from the percentage of women with HIV on ART in Uganda [38] and reflects Uganda Ministry of Health guidelines that were in place at the time of the study, which did not recommend TLD as a preferred regimen for women of childbearing potential [6]. This guideline has since changed to recommend TLD for all adults [7], and thus TLD usage is now much more common among women than at the time of our study, although still lagging in the region [39, 40]. Loss to follow-up was also rare in this study, occurring in only 1% of participants. This could be attributable to phone and home tracking procedures utilized in this study, which may not be feasible in routine care, although of note, similarly low rates of loss to follow-up have been reported previously at this site [12, 14]. In addition, patient-reported outcomes on satisfaction with care and health-related quality of life were not collected in this study but are being planned in future projects to better understand drivers of retention in care in this population.

In this study, 5% of the cohort did not achieve viral suppression with retention in care due to virologic failure, death, or loss from care, which could correspond to a high number of individuals with adverse clinical outcomes in settings with a high prevalence of HIV. Thus, ongoing vigilance and targeted efforts are needed to optimize adherence and retention in care to bolster the long-term success of TLD in the region. However, the high rates of viral suppression observed in this cohort, coupled with no observed treatment-emergent resistance to DTG and a low TLD discontinuation rate, support the global policy shift toward implementation of TLD as the preferred ART regimen in resource-limited settings.

## Supplementary Data

Supplementary materials are available at *The Journal of Infectious Diseases* online (http://jid.oxfordjournals.org/). Supplementary materials consist of data provided by the author that are published to benefit the reader. The posted materials are not copyedited. The contents of all supplementary data are the sole responsibility of the authors. Questions or messages regarding errors should be addressed to the author.

## Supplementary Material

jiae260_Supplementary_Data

## Notes

***Acknowledgements.*** We thank our study participants for their time and dedication to the mission of research and acknowledge the staff of the Mbarara Regional Referral Hospital Immune Suppression Syndrome Clinic for their support of our study. We dedicate this work in memory of Dr Mwebesa Bosco Bwana, a gifted mentor, compassionate physician, dedicated researcher, and friend to many who was a site principal investigator for this study until his passing in 2020.

***Author contributions.*** S. M. M., W. M., B. H. G., V. C. M., M. Y. M., D. P., R. K. G., and M. J. S. contributed to the study design. S. M. M., W. M., V. N., D. O., N. K., I. B., L. K., and J. T. oversaw data and specimen collection. D. O. processed the laboratory specimens for the study and conducted the viral load assays. R. L. conducted the genotypic resistance testing and resulting bioinformatics for the study. S. M. M., J. T., T. N. A., and A. S. curated the study data and conducted data quality checks. S. M. M. conducted the data analysis and drafted the manuscript. T. N. A. generated tables and figures for the manuscript. B. H. G. and M. J. S. provided consultation and oversight of the statistical analysis. All authors contributed to critical review of the manuscript.

***Disclaimer***. The contents are solely the responsibility of the authors and so not necessarily represent the official views of the National Institutes of Health.

***Data availability.*** Data can be made available upon request to the corresponding author.

***Financial support*.** This work was supported by the National Institutes of Health (grant number K23 AI143470 to S. M. M.); ViiV Healthcare (investigator-initiated grant number 212215 to M. J. S.); and Wellcome Trust (grant number WT108082AIA to R. K. G.). M. J. S. received additional support from the National Institutes of Health (grant number K24 HL166024) and V. C. M. from the Emory Center for AIDS Research (grant number P30AI050409).
