# Supplementary material for: Population Effectiveness of Dolutegravir Implementation in Uganda: A Prospective Observational Cohort Study (DISCO), 48-Week Results
Source: J Infect Dis. 2024 May 16;230(3):e622–30. doi: 10.1093/infdis/jiae260 (PMC11420783; doi:10.1093/infdis/jiae260)
Supplement: jiae260_Supplementary_Data [file jiae260_supplementary_data.docx]

| **Supplementary Table 1: Multivariable logistic regression model to assess predictors of viral suppression defined as <50 copies/mL after one year of follow-up in the DISCO Cohort** | | | | |
| --- | --- | --- | --- | --- |
| **Predictors** | **Proportion** | **AOR** | **95% CI** | **P value** |
| HIV-1 RNA viral load at time of switch to TLD |  |  |  |  |
| <50 copies/mL at the time of switch | 441/475 | Reference |  |  |
| ≥50 copies/mL at the time of switch | 17/24 | 0.12 | 0.04-0.33 | <0.001 |
| Gender |  |  |  |  |
| Female | 196/205 | Reference |  |  |
| Male | 263/295 | 0.38 | 0.16-0.91 | 0.030 |
| Self-reported adherence during follow-up |  |  |  |  |
| Adherence ≥90% | 416/442 | Reference |  |  |
| Any adherence <90% | 43/51 | 0.28 | 0.12-0.69 | 0.006 |
| Abbreviations: TLD, tenofovir/lamivudine/dolutegravir; CI, Confidence Interval; AOR, Adjusted Odds Ratio | | | | |
